# Characterisation of maturation of photoreceptor cell subtypes during zebrafish retinal development

**DOI:** 10.1242/bio.036632

**Published:** 2018-09-20

**Authors:** Cátia Crespo, Elisabeth Knust

**Affiliations:** Max-Planck-Institute of Molecular Cell Biology and Genetics, Pfotenhauerstrasse 108, 01307, Dresden, Germany

**Keywords:** Mitochondria, Nuclei, Polarity, Light, Growth, Chromatin

## Abstract

Photoreceptor cells (PRCs) mature from simple epithelial cells, a process characterised by growth and compartmentalisation of the apical membrane into an inner and an outer segment. So far, a PRC subtype-specific description of morphological and cellular changes in the developing zebrafish retina is missing. Here, we performed an in-depth characterisation of four of the five PRC subtypes of the zebrafish retina between 51 and 120 h post fertilisation, including quantification of the size of different compartments, localisation of polarity proteins and positioning of organelles. One of the major findings was the anisotropic and subtype-specific growth of the different PRC compartments. In addition, a transient accumulation of endoplasmic reticulum in rod PRCs, changes in chromatin organisation in UV sensitive cones and differential expression of polarity proteins during the initial stages of PRC maturation were observed. The results obtained provide a developmental timeline that can be used as a platform for future studies on PRC maturation and function. This platform was applied to document that increased exposure to light leads to smaller apical domains of PRCs.

## Abstract (precis)

**Summary:** We characterised subtype-specific growth of the different photoreceptor compartments, organelle distribution and the influence of light on the growth of the apical membrane.

## INTRODUCTION

Photoreceptor cells (PRCs) are highly polarised neurons present in the retina, which are specialised for phototransduction. Polarity is manifested by the subdivision of the plasma membrane into an apical and a basal side, separated by cell junctions, which define the so-called outer limiting membrane (OLM) (Zou et al., 2012). The basal side comprises the synaptic terminals of the PRCs, which transmit the light-induced visual signals to the post-synaptic retinal network (Regus-Leidig and Brandstätter, 2012). The apical portion of PRCs is subdivided into two distinct compartments, the inner segment (IS) and the outer segment (OS) (Roepman and Wolfrum, 2007). The OS is a modified primary cilium characterised by stacks of membranous discs containing the visual pigment, which is responsible for absorption of photons (Steinberg et al., 1980). The visual pigment is composed of opsin, a seven-transmembrane domain protein of the G-protein-coupled receptor (GPCR) superfamily, and a covalently bound chromophore, retinal (Samardzija et al., 2009; Suliman and Novales Flamarique, 2014; Yokoyama, 2000). The IS contains the organelles necessary for protein and lipid biosynthesis and energy production of the cell and can be divided into two different regions in adult PRCs. The most apical area, called the ellipsoid, is characterised by clustered mitochondria. The region between the mitochondria and the nucleus is called the myoid, which contains the Golgi apparatus and the endoplasmic reticulum (ER) (Papermaster et al., 1985; Tarboush et al., 2014). The IS can further be distinguished from the outer segment by the localisation of several polarity proteins, such as Crb2a, Crb2b or the atypical protein kinase C proteins, PrkCi and PrkCz (Krock and Perkins, 2014; Omori and Malicki, 2006; Zou et al., 2012). These proteins are also essential for the establishment and maintenance of apico-basal polarity of PRCs. Knockdown or overexpression studies revealed their importance for specification of the apical membrane, epithelial tissue integrity and proper lamination of the retina (Cui et al., 2007; Krock and Perkins, 2014; Malicki and Driever, 1999; Omori and Malicki, 2006).

Vertebrate PRCs are classified into two major subtypes: rods, which are more sensitive to light and important for dim light vision, and cones, essential for day light and colour vision. Cone PRCs in the zebrafish retina are further subdivided into four classes, based on the expression of different opsin proteins: SWS (short wave or blue-sensitive) cones, LWS (long wave or red-sensitive) cones, MWS (middle wave or green-sensitive) cones and UVS (ultraviolet-sensitive) cones (Chinen et al., 2002; Vihtelic et al., 1999). Based on their morphology, cone PRCs can additionally be divided into two categories: single (UVS or SWS cones) and double cones (MWS, LWS cones) (Branchek and Bremiller, 1984).

The zebrafish retina develops in a wave-like manner from a simple, undifferentiated epithelium into a complex sensory organ, which is organised into well-defined layers (Almeida et al., 2014; Neumann, 2001; Schmitt and Dowling, 1999). The PRC layer first appears in the ventral patch at about 50 h post fertilisation (hpf). Although functionally mature PRCs have a complex morphology as described above, they arise from relatively simple columnar precursors (Weber et al., 2014). Previous transmission electron microscopy studies on the zebrafish retina first detected OSs on the ventral patch at 55 hpf (Kljavin, 1987; Raymond et al., 1995; Schmitt and Dowling, 1999). At the same time, the positioning the different organelles allow a clear distinction between the ellipsoid and myoid regions of the IS (Schmitt and Dowling, 1999). At 96 hpf, two different types of cone PRCs can be distinguished, based on cellular morphology and position within the PRC layer. One cell-type resembles the adult single cone PRCs (UVS or SWS cones), whereas the other resembles the adult double cone PRCs (LWS and MWS cones). By 12 days post fertilisation (dpf), all PRC subtypes can be identified. Cone PRCs achieve their full adult dimensions at 15 dpf, in comparison to rod PRCs, which are fully matured at 20 dpf (Branchek and Bremiller, 1984; Hitchcock and Raymond, 2004; Raymond et al., 1995). In the mature zebrafish retina, UVS cones are the shortest PRCs, while SWS cones are of intermediate size, and double cones, comprised of two non-identical cones adhered together (MWS and LWS cones), are the longest (Branchek and Bremiller, 1984).

In the past, several studies on PRC polarity establishment, differentiation and maturation have been performed in the zebrafish retina, which provided a more global view of the processes taking place along the whole retina (Hamaoka et al., 2002; Krock and Perkins, 2014; Omori and Malicki, 2006; Raymond et al., 1995; Schmitt and Dowling, 1999). However, a detailed characterisation of the maturation of the different PRCs subtypes, including subtype-specific differentiation of the apical membrane into a photosensitive structure, cellular growth, or organelle distribution is missing. This information would be particularly useful to unravel in more detail the genetic or environmental influences on specific PRC subtypes, and thus could ultimately also contribute to our understanding of the origin of retinal diseases.

In this study, we characterised the maturation stages of PRC subtypes in the zebrafish retina from 51 hpf to 120 hpf. The analysis included quantification of growth of the different PRC compartments, localisation of polarity proteins and distribution of different organelles. The results obtained not only allow us to identify subtype-specific features from very early stages onwards, but also provide novel features to determine maturation stages of PRCs development, independent of the age of the developing embryo. The latter is crucial, since the zebrafish retina develops in a wave-like manner (Almeida et al., 2014), where different regions of the retina contain PRCs at different maturation stages, and most zebrafish mutant lines exhibit delayed and/or defective development, which precludes precise staging by embryonic age. We finally applied this knowledge to show that light negatively affects the growth of the IS and OS of LWS cones at late stages of maturation.

## RESULTS

### Different compartments of photoreceptor cells show a dynamic growth pattern

**Fig. 1.**
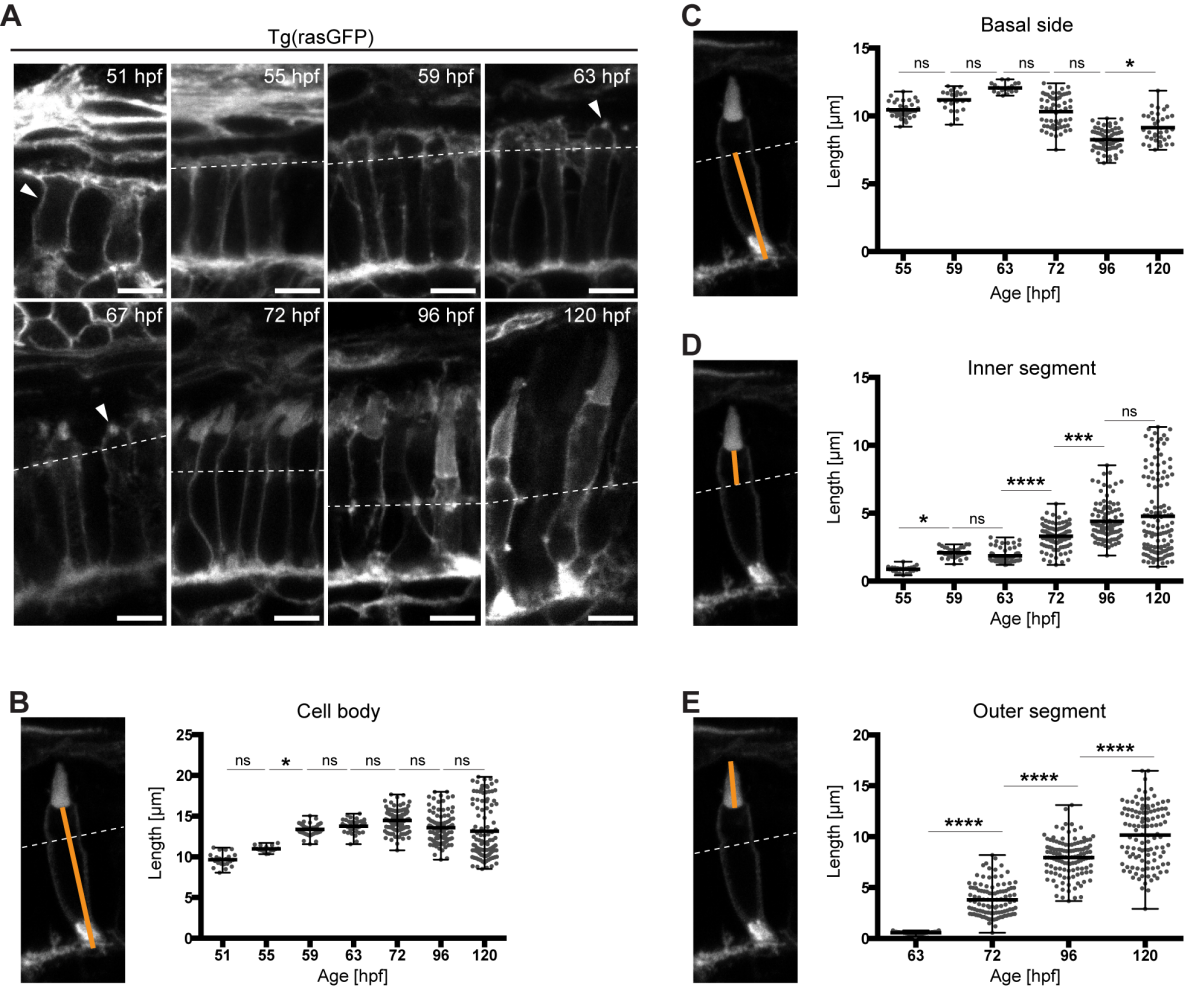
**Growth of the different PRC compartments during maturation.** (A) Confocal images of the PRC layer in retinal sections of Tg(rasGFP) embryos, with plasma membrane in grey at various timepoints. hpf: hours post fertilisation. Dashed lines mark the level of the outer limiting membrane, OLM. At 51 hpf, the arrowhead outlines a PRC precursor and at 63–67 hpf an emerging outer segment (OS). Scale bars: 5 µm. (B–E) Confocal images of individual PRCs from retinal sections of Tg(rasGFP) animals at 72 hpf. The orange lines mark the compartment measured. The quantification of the length of different PRC compartments at different developmental times are shown adjacent to each image. Length of cell body (B), basal side (C), inner segment (D) and outer segment (E) are represented in dot plots with calculated minimum, mean, and maximum. On average, 70 cells from a total of 4–6 independent embryos were quantified per timepoint. Statistical significance was calculated by a one-way ANOVA followed by Tukey's multiple comparison test. The significance is as follows: ns (non-significant)=*P*>0.05, *=*P*≤0.05, **=*P*≤ 0.01, ***=*P*≤0.001, ****=*P*≤0.0001.

Photoreceptor cells (PRCs) arise from polarised columnar epithelial cell precursors. During development cells grow, and in particular in the apical plasma, which becomes further subdivided into the inner and outer segment (IS and OS). To monitor changes in cell morphology and compartment growth during PRC maturation, we used the transgenic line Tg(Ola.Actb:Hsa.HRAS-EGFP)^vu119^ (Cooper et al., 2005), in which the plasma membrane of all cells is labelled with GFP (Fig. 1A). This transgenic will be referred to as Tg(rasGFP) from now on. This line is suitable to distinguish various membrane compartments, such as the adherens junctions, which show co-localisation of transgene-encoded GFP and ZO-1 or actin (Fig. S1B). In later stages, this region is defined as the outer limiting membrane (OLM) (Fig. 1A, dotted line; Fig. S1C). At 72 hpf (hours post fertilisation), transgene-encoded GFP additionally marks the OS, as revealed by co-localisation with opsin (Fig. S1D). Therefore, the use of Tg(rasGFP) allows us to distinguish the various cellular compartments, i e. the IS, OS and basal membrane, and thus enables the quantification of the growth of the different compartments.

To do so, a time-course was established, starting at 51 hpf with the emergence of committed PRC precursors, and finishing at 120 hpf. For this time-course, samples were taken at 4 h intervals between 51 and 72 hpf, when the majority of cells become post-mitotic (He et al., 2012), and at 1 d intervals from 72 hpf onwards (51 hpf, 55 hpf, 59 hpf, 63 hpf, 67 hpf, 72 hpf, 96 hpf and 120 hpf). We focused on timepoints up to 120 hpf, because by 120 hpf all PRCs show their characteristic compartmentalisation (Branchek and Bremiller, 1984). In addition, accurate measurements of PRC OSs are no longer possible at later timepoints due to the growth of the retinal pigmented epithelium. In order to compare different timepoints, all images were taken from an area between the optic nerve and the ventral patch (Fig. S1A). This is essential because the retina differentiates in a wave, and different stages of PRC developmental can be observed within the same retina (Almeida et al., 2014; Schmitt and Dowling, 1999).

At 51 hpf, the region of interest (Fig. S1A) consists of columnar epithelial cells, with the adherens junctions localised apically, separating the apical and basolateral membranes (Fig. 1A; Fig. S1B). Some cells show a round morphology, characteristic of cells in mitosis (arrow in Fig. S1B). Since in fixed tissue it is not possible to define whether a cell has exited the cell cycle, all columnar cells present in the PRC layer exhibiting a simple epithelial structure, i.e. without any obvious subdivision of the apical membrane, will be referred to as PRC precursors. The cell body of PRC precursors varies in size from 8.1 µm to 11.1 µm at about 51 hpf, with an average size of 9.7 µm (Fig. 1B). Cell body length was measured from the mid-point of the basal membrane to the midpoint of the apical membrane in PRCs with no OS, and from the midpoint of the basal membrane to the midpoint of the base of the OS in cells showing an OS. From 51 hpf to 55 hpf, no significant change in cell body length was observed (Fig. 1B).

Between 55 and 59 hpf, IS length and cell body length increased (Fig. 1B,D). However, no growth of the basal side occurred during this period (Fig. 1C). From this we conclude that the overall increase in cell body length is mostly due to IS growth. No significant difference was observed in IS and cell body length between 59 and 63 hpf (Fig. 1B,D). At these stages, processes extending from the IS were observed both in confocal images and electron micrographs (Fig. S2), the nature and importance of which are not understood. For the remaining timepoints, no increase in the average cell body length was observed (Fig. 1B), while the IS length increased gradually (Fig. 1D).

OSs were evident by immunohistochemistry in some cells at 63 hpf and in all cells by 67 hpf (Fig. 1A, arrow head). Since no obvious differences in cell morphology were observed between 63 hpf and 67 hpf, all quantifications of growth were done at 63 hpf only (Fig. 1). From 63 hpf to 96 hpf, a decrease in the basal side was observed. From 72 hpf onwards OS size continuously increased in a more linear way while ISs average length only shows a small increase up to 96 hpf. However, for both ISs and OSs the variability increased dramatically, as well as the maximum values obtained (Fig. 1D,E). Surprisingly, the minimum values of IS length by 120 hpf show a small decrease when compared to 96 hpf, indicating a decrease of IS length in a subset of PRCs (Fig. 1D). The high variability in values in both IS and OS length is most likely due to differential growth in the different PRC subtypes. By 120 hpf, ISs and OSs reached an average length of 4.8 and 10.2 µm, respectively (Fig. 1D,E).

### Different PRC subtypes show differential compartment growth

**Fig. 2.**
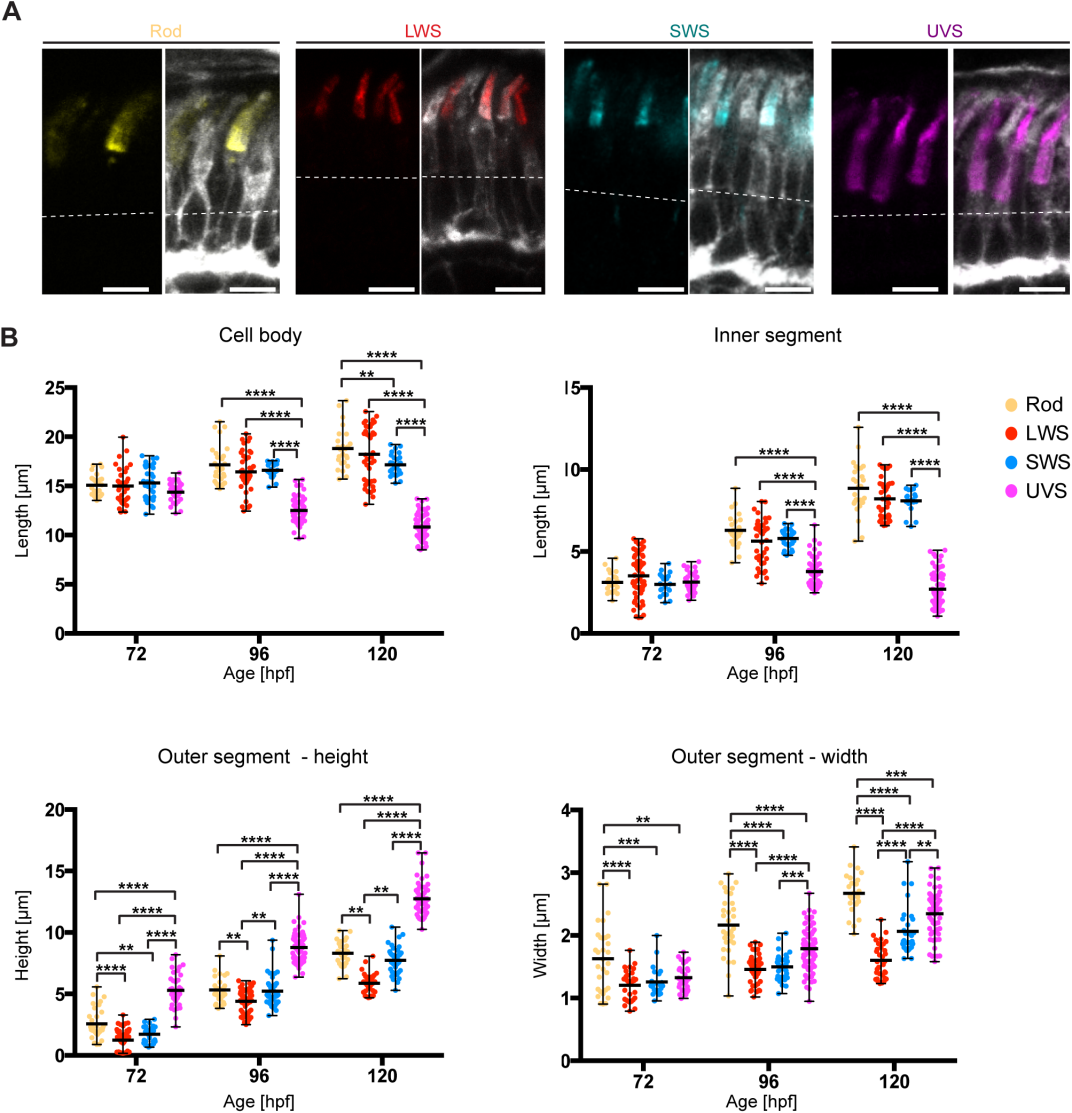
**PRC subtypes show different growth of the apical domain.** (A) Confocal images of the PRC layer in retinal sections of Tg(rasGFP) embryos at 120 hpf, with plasma membrane in grey and antibody staining for different opsins: rhodopsin (yellow), red opsin (red), blue opsin (blue) and UV opsin (magenta). Dashed lines mark the level of the OLM. Scale bars: 5 µm. (B) Length of the cell body, the inner and the outer segment height and width at various stages are represented in dot plots with calculated minimum, mean, and maximum. On average, 40 cells from a total of 6–8 independent embryos were quantified per timepoint. Statistical significance was calculated by a two-way ANOVA followed by Tukey's multiple comparison test. Within the same time-point, all PRCs show no significant difference (*P*>0.05), with exception of samples highlighted with **(*P*≤0.01), ***(*P*≤0.001) and ****(*P*≤0.0001).

In order to investigate the growth of the different PRC subtypes we used Tg(rasGFP) retinal sections stained with different opsin antibodies (rhodopsin, UV, red and blue) (Fig. 2A), and measured compartments length at the timepoints when the largest variability in values was previously observed, namely 72 hpf, 96 hpf and 120 hpf. Unfortunately, MWS cones could not be identified, since an antibody against green opsin was not available.

In accordance with the literature, obvious differences in morphology were evident from 96 hpf onwards (Branchek and Bremiller, 1984). At 96 hpf UVS cones became clearly distinguishable by their small ISs. These differences became more pronounced by 120 hpf (Fig. 2A). To quantify the growth of the different compartments of PRC subtypes, we measured the cell body and IS length and OS height. In addition, OS width was quantified by measuring the length of the OS base. For this quantification, cells from both ventral and basal sides of the optic nerve were analysed, excluding cells from the ventral patch, which are in more advanced stages of maturation.

At 72 hpf no statistically significant differences in IS and cell body length were detected among PRC subtypes (Fig. 2B). Obvious differences were detected at 96 hpf and increased over time between UVS cones and other PRCs. However, no significant difference was found among the other PRC subtypes in terms of IS length. Between 72 and 120 hpf rods and LWS cones showed significant cell body increase, with an average increase of 25% and 21%, respectively (Table 1). In contrast, UVS cones showed an average decrease of approximately 25% (Table 1). Similarly, the average length of the IS of UVS showed a decrease of 13%, and all other PRC subtypes showed an increase of 134–187%. At 120 hpf UVS cones showed the smallest cell body and IS, followed by the SWS cones, LWS cones and finally rods (Fig. 2B). These data suggest that, with the exception of UVS cones, PRC subtypes show a comparable IS and cell body increase over time.

**Table 1.**
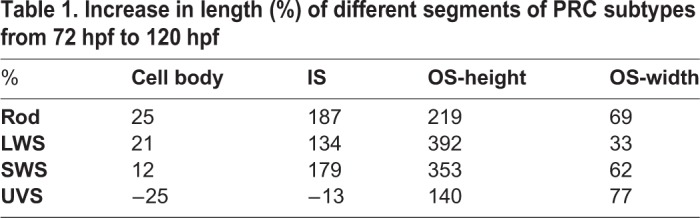
**Increase in length (%) of different segments of PRC subtypes from 72 hpf to 120 hpf**

Contrary to the cell body and the IS length, PRC subtypes showed obvious differences in OS height and width already at 72 hpf. Interestingly, from an early stage onwards, UVS cones showed the highest average in OS height with 5.3 µm (Fig. 2B), while LWS and SWS cones showed the smallest average heights, with 1.2 µm and 1.7 µm, respectively. At this stage, OS width of cone PRC subtypes showed no significant differences (1.2–1.3 µm). Rods, on the other hand, showed the greatest width, with an average of 1.6 µm (Fig. 2B), which will further increase over time.

From 72 hpf to 120 hpf, the OS is the cell compartment with the highest increase in length in all PRC subtypes (140–392% in OS height and 33–77% in OS width) (Table 1). Additionally, by 120 hpf significant differences in OS height and width can be observed among all different types of PRCs. By 120 hpf, LWS cones showed the lowest height (5.9 µm), followed by SWS cones (7.7 µm), rods (8.3 µm) and finally UVS cones (12.7 µm) (Fig. 2B). By 120 hpf rod PRCs are the widest at 2.7 µm, followed by UVS cones at 2.3 µm, SWS cones at 2.0 µm, and finally LWS cones at 1.6 µm (Fig. 2B).

To summarise, cone PRCs with larger OS height also show larger OS width. In addition, differences in OS size exist not only between rod and cone PRCs, but also among cone PRC subtypes. Surprisingly, smaller IS length correlated with larger OS length. These data highlight the importance to carefully distinguish PRC subtypes when analysing PRC growth for timepoints later than 72 hpf.

### Polarity proteins show differential localisation at early stages of PRC maturation

In zebrafish, the polarity gene *crb2a* (*oko meduzy, ome*) and *crb2b* were shown to have an impact on apical growth of PRCs (Hsu and Jensen, 2010; Omori and Malicki, 2006). Localisation of these two proteins during the initial stages of PRC maturation has not yet been investigated. Therefore, we stained retinal sections of Tg(rasGFP) embryos with antibodies specific for Crb2a and Crb2b. In addition, we used an antibody against both PrkCi and PrkCz, another apical marker, to better follow the development of polarity during early stages of PRC maturation (between 51 and 63 hpf). From now on we will refer simply to PrkC when referring to the combination of both PrkCi and PrkCz.

**Fig. 3.**
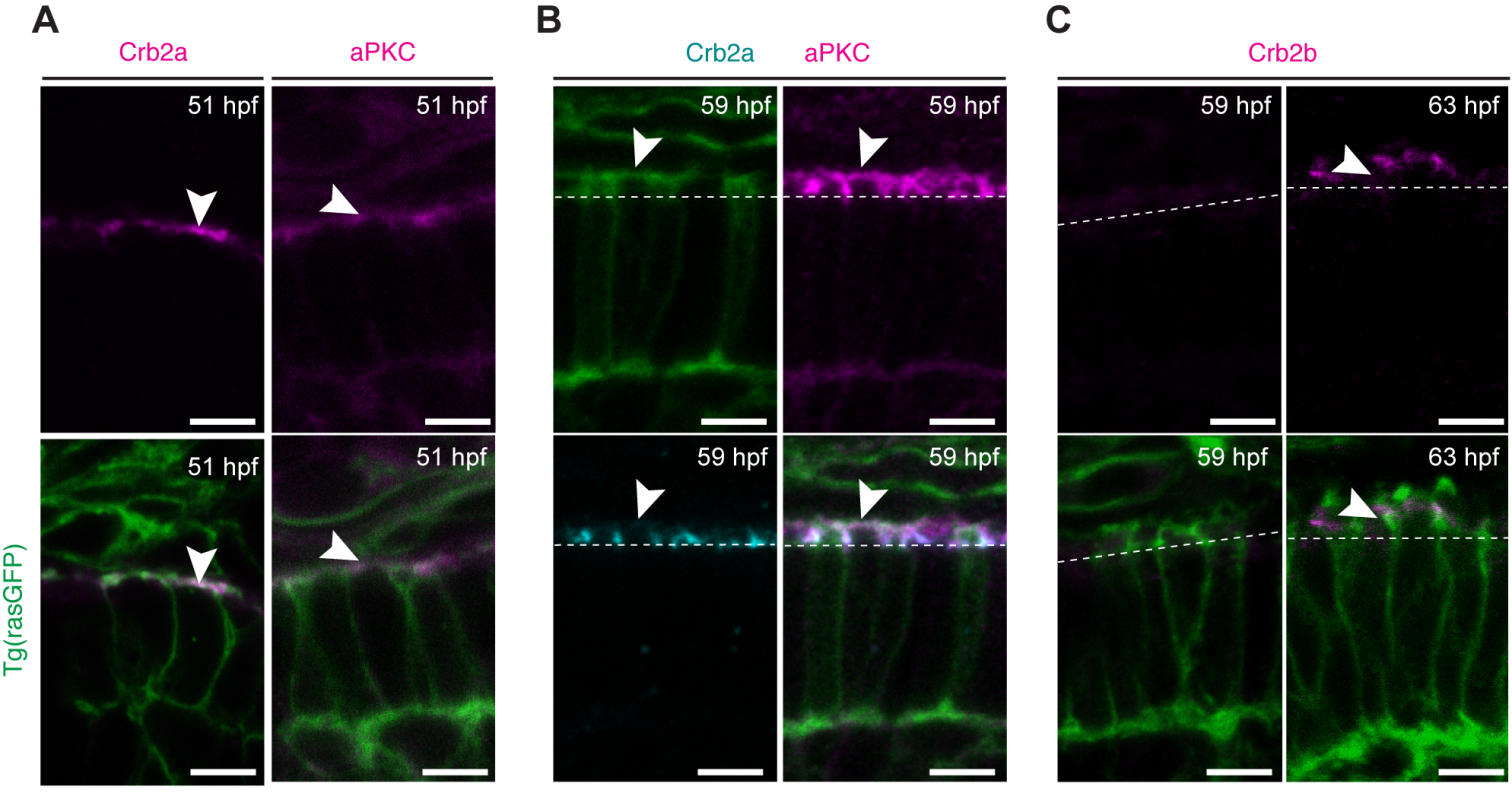
**Polarity proteins show differential expression at the initial stages of PRC maturation.** Confocal images of the PRC layer in retinal sections of Tg(rasGFP) embryos showing the plasma membrane in green and the respective polarity protein. (A) Crb2a and PrkC antibody staining of embryos at 51 hpf in magenta. (B) PrkC (magenta) and Crb2a (cyan) antibody staining of embryos at 59 hpf. (C) Crb2b antibody staining of embryos at 59 hpf and 63 hpf in magenta. Dashed lines mark the level of the OLM and arrowheads highlight antibody staining. Scale bars: 5 µm.

At 51 hpf, both Crb2a and PrkC are localised to the entire apical membrane of PRC precursors (Fig. 3A). With the formation of the IS at 55 hpf, Crb2a becomes restricted to the lateral membrane of the IS, defining the SAR (subapical region), and was undetectable in the most apical membrane (Fig. 3B). In contrast, PrkC was detected both at the free apical membrane and the SAR (Fig. 3B). Unlike Crb2a and PrkC, Crb2b could not be detected when the IS first emerged (55–59 hpf) (Fig. 3C). By 63 hpf, Crb2b could be detected at the SAR of some PRCs (Fig. 3B). Based on the literature (Zou et al., 2012), we trust these PRCs to be LWS, SWS and MWS cones. These data point to a dynamic expression and localisation of polarity proteins in the initial stages of PRC maturation, suggesting specific functions for each of them. Additionally, this difference in localisation could be used as a marker for more precise staging of maturing PRCs.

### PRC subtypes show differential organelle organisation

An additional tool to follow maturation of PRCs is to follow the distribution of different organelles, particularly at later stages. Therefore, we studied various organelles by confocal and transmission electron microscopy (TEM) in embryos from 51 hpf to 120 hpf in order to gain better insights on organelle distribution and ultrastructure.

**Fig. 4.**
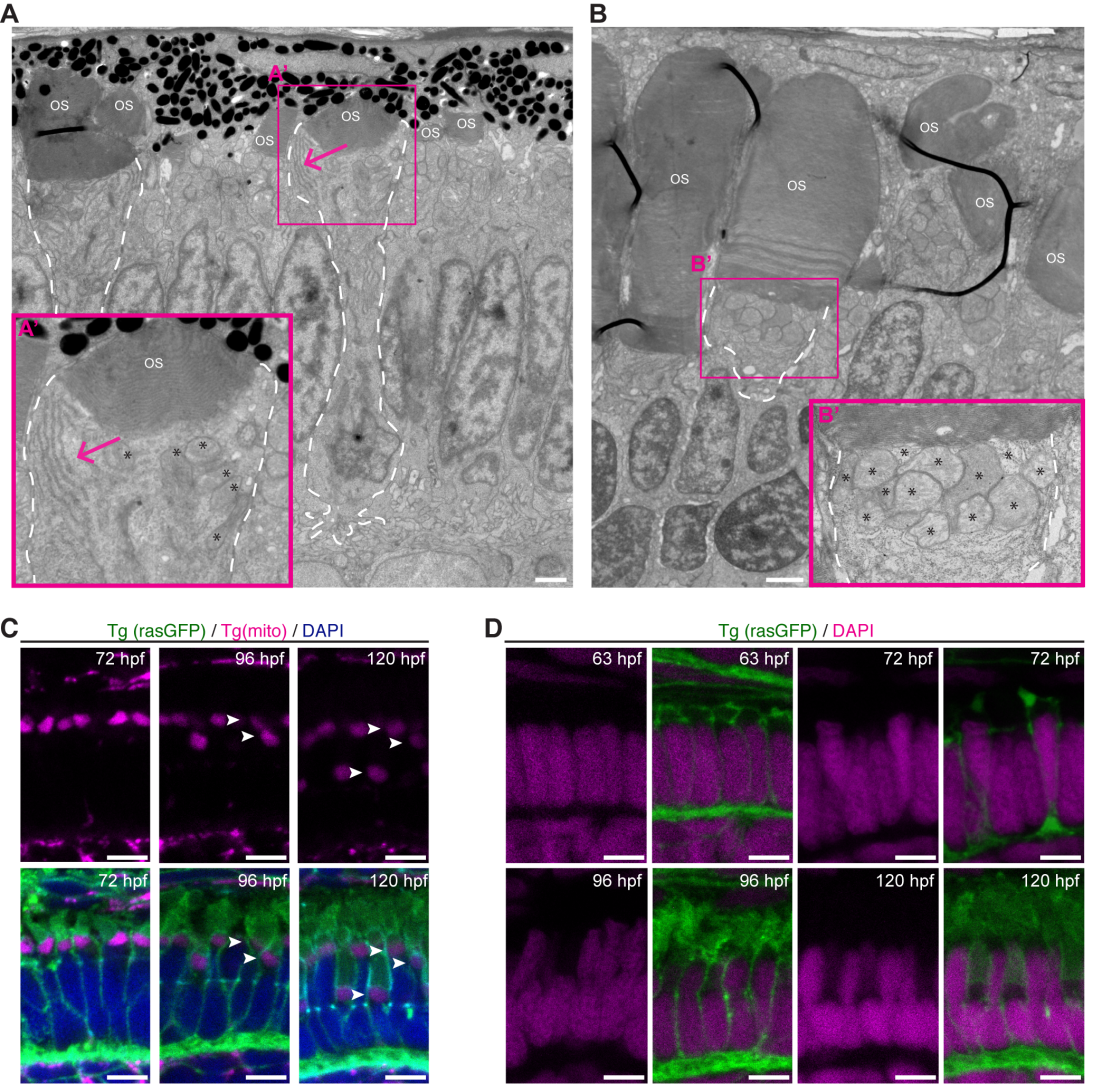
**Organelles show different positioning during PRC maturation.** (A) Electron micrograph of a section through the PRC layer of a wild-type zebrafish embryo at 72 hpf. (A′) Blow-up of the box indicated in A, showing Endoplasmic reticulum (ER) clusters (arrow) in the ellipsoid region of rod-like PRCs. (B) Electron micrograph of a section through the PRC layer in the ventral patch, a rod enriched region, of a wild-type zebrafish embryo at 120 hpf. (B′) Blow-up of the box indicated in B, showing mitochondria accumulate in the inner segment (arrow). ER clusters are no longer visible. For both A and B, asterisks mark the mitochondria, ‘OS’ the OSs, and PRCs with large OS are outlined with a dashed line. (C) Confocal images of retinal sections of zebrafish embryos at 72 hpf, 96 hpf and 120 hpf. Endogenous fluorescence of Tg(mito:GFP) (magenta) and Tg(rasGFP) (green). DAPI staining (nuclei) in blue. Arrowheads point to mitochondria. Note that mitochondria can be found in three distinct layers by 120 hpf. (D) Confocal images of retinal sections of zebrafish embryos at 63 hpf, 72 hpf, 96 hpf and 120 hpf. Endogenous fluorescence of Tg(rasGFP) (green) DAPI staining in magenta. Scale bars: A–B: 1 µm, C–D: 5 µm.

**Fig. 5.**
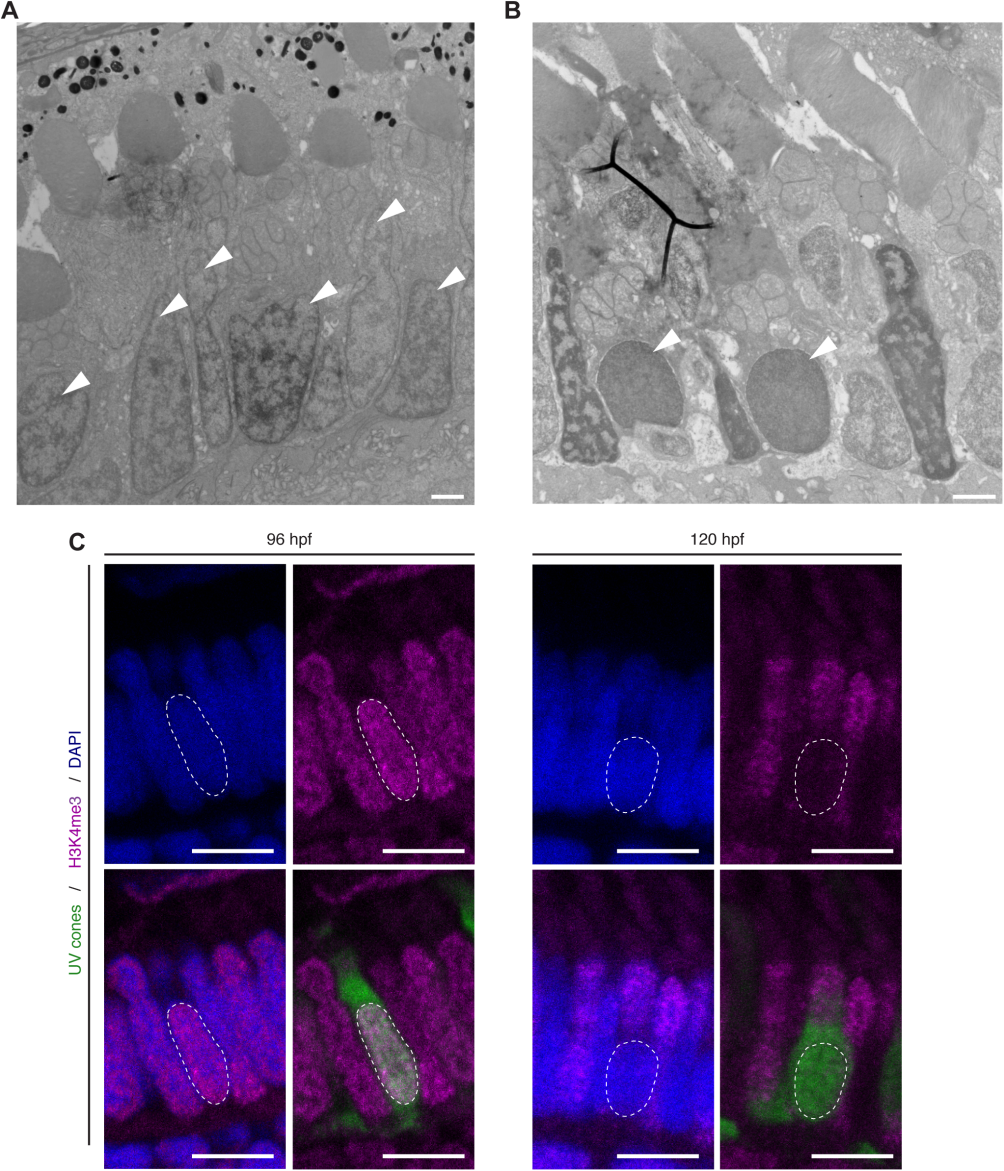
**UV sensitive cones show changes in chromatic organisation.** (A,B) Electron micrographs of sections through the PRC layer in the central retina of a wild-type zebrafish embryos at 96 hpf (A) and 120 hpf (B). Nuclei of some PRCs show alteration in chromatin at 120 hpf (arrowheads). (C) Confocal images of the PRC layer in retinal sections of Tg(-5.5opn1sw1:EGFP) embryos at 96 hpf and 120 hpf, with DAPI in blue, antibody staining for H3K4me3 in magenta and endogenous GFP fluorescence in green. UVS positive cells (green) show no staining for H3K4me3, a marker for euchromatin. Dashed lines mark the nucleus outline of a UVS cone. Scale bars: A–B: 1 µm, C: 5 µm.

By 72 hpf a subset of PRCs across the retina show large accumulations of rough ER (endoplasmic reticulum) in the ellipsoid region (Fig. 4A), which is in agreement with previously published data (Kljavin, 1987). However, by 120 hpf this large accumulation of rough ER can no longer be detected at the ellipsoid region (Fig. 4B). Localisation of ER in the ellipsoid at 72 hpf was always associated with an OS width of at least 2.0 μm, suggesting these cells to be rods. This assumption is corroborated by the fact that this ER accumulation is present in most cells at the ventral patch (Fig. 5B), an area enriched with rods (Schmitt and Dowling, 1999).

As revealed by transmission electron microscopy, mitochondria of PRCs in zebrafish older than 96 hpf are localised in the IS (Tarboush et al., 2014). At 55 hpf, before OS can be detected by TEM, mitochondria are localised to the apical region of PRCs, however, no distinction between myoid and ellipsoid regions is yet possible at this timepoint (Fig. S2). Additionally, we used Tg(mito:GFP), a transgenic line that tags mitochondria with GFP, to follow mitochondrial localisation during later stages of PRC maturation. At 72 hpf, the mitochondrial clusters in all cells are aligned in a single row (Fig. 4C). However, by 96 hpf, two rows of mitochondria can be identified, and by 120 hpf, three rows of mitochondria layers could be observed (Fig. 4C). These changes are due to the different positioning of the IS of distinct PRC subtypes within the PRC layer.

Another potential marker for PRC maturation is positioning of PRC nuclei. The nuclei of PRC subtypes were reported to localise at different levels in the outer nuclear layer from early stages of development onwards (Fang et al., 2017; Kljavin, 1987). To better understand changes in nuclear positioning during PRC maturation, we used confocal microscopy to visualise nuclei stained with DAPI, and TEM. Nuclei of zebrafish embryos up to 63 hpf showed a rectangular shape and no differences could be observed among different PRC subtypes (Fig. 4D). However, from 72 hpf onwards, nuclei start to occupy different levels. At 96 hpf, two independent layers of nuclei were obvious, which became more pronounced in PRCs at 120 hpf (Fig. 4D).

When studying nuclei positioning by TEM, we noticed changes in chromatin organisation in a subset of PRCs at later stages of development. In some vertebrates, such as mouse, rods were shown to have differences in chromatin organisation, which could be essential for PRC function (Solovei et al., 2009). In order to understand if the differences in chromatin organisation observed in zebrafish are PRC subtype-specific, we studied the retina of embryos at 72 hpf, 96 hpf and 120 hpf by TEM. Up to 96 hpf, all nuclei showed a similar distribution of chromatin (Fig. 5A), with dark staining enriched at the nuclear envelope and in irregular clusters, probably representing heterochromatin, and lighter staining in the nuclear interior between heterochromatin compartments, probably representing euchromatin. Surprisingly, by 120 hpf a subset of cells showed a more homogeneous distribution of chromatin (arrow in Fig. 5B). Based on cell morphology and mitochondria positioning, cells with modified chromatin organisation are likely to be UVS cones.

To corroborate this assumption, we performed antibody stainings for H3K4me3, a marker for euchromatin, in the [Tg(-5.5opn1sw1:EGFP) transgenic line (Takechi et al., 2003)], which marks the cytosol of UVS cones with GFP (Fig. 5C). At about 96 hpf, all nuclei have a similar distribution of euchromatin. However, at 120 hpf, no antibody staining for H3K4me3 could be detected in nuclei (stained by DAPI) of GFP positive cells (Fig. 5C), which is in accordance with the TEM data described above (Fig. 5A,B). Our results are the first to show a change in chromatin organisation in nuclei of the zebrafish retina, and that these changes seem to be specific for UVS cones.

Taken together, these results demonstrate that organelle distribution and chromatin organisation can be used as a novel and more precise way to stage PRC maturation and to help distinguishing the different subtypes.

### Apical expansion of PRC during maturation is dependent on light exposure

**Fig. 6.**
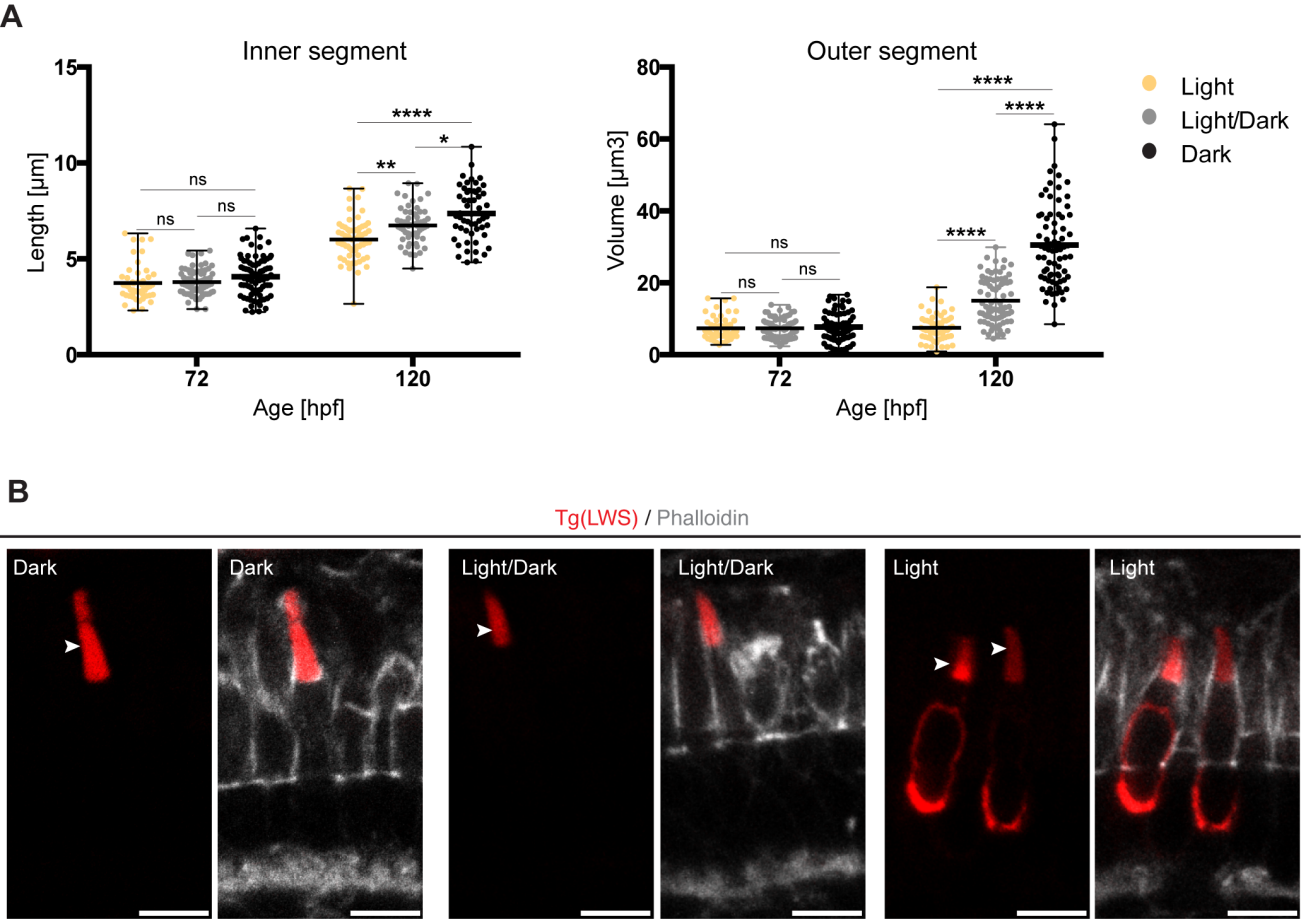
**Exposure to constant light prevents leads to small apical domains in maturing PRCs.** (A) IS length and OS volume of LWS cones of fish kept at different light conditions are shown: constant light (yellow), light/dark cycle (grey) and constant dark (black). All measurements are represented in dot plots with calculate minimum, mean, and maximum. On average, 65 cells from 4–6 independent embryos were quantified per timepoint. Statistical significance was calculated by a one-way ANOVA followed by Tukey's multiple comparison test. The significance is as follows: ns (non-significant) =*P*>0.05, *=*P*≤0.05, **=*P*≤ 0.01, ****=*P*≤0.0001. (B) Confocal images of retinal sections of Tg(LWS) zebrafish embryos at 120 hpf. Embryos were exposed to different light conditions, constant dark, light/dark cycle (14 h/10 h) and constant light. mKate2-tagged opsin is shown in red and phalloidin staining in grey. Outer segments are highlighted by arrowheads. Scale bars: 5 µm.

The differential growth behaviour of individual PRC compartments, combined with the tools that allow to more precisely determine the developmental stage and subtype of maturing PRCs presented above, puts us into a much better position to study the impact of the genetic background and environmental influences on PRC maturation. Here, we concentrated on the role of light on the growth behaviour of PRCs. It has been shown that light exposure results in a decrease of OS size of human PRCs (Abràmoff et al., 2013), and in changes of the positioning of the myoid in adult zebrafish PRCs (Hodel et al., 2006). However, no data exist so far that show any influence of light on PRC growth during embryonic zebrafish retinal development. Therefore, we compared OS volumes and IS lengths of embryos raised either in the dark, in a dark/light cycle (10 h dark/14 h light), or in constant light at 72 hpf and 120 hpf. To avoid variability due to the different developmental stages present in the retina, we focused on cells at the ventral and dorsal side of the optic nerve, with the exception of the ventral patch. Additionally, we used positioning of nuclei and ER as described above as markers for PRC maturation. No changes in these parameters were observed under the different light conditions (data not shown), showing that there was no overall delay in retinal development and apical differentiation. Due to the size variability observed when globally measuring all PRCs (Fig. 1), we focused the analysis on LWS cones, one of the most abundant PRC subtypes in the zebrafish retina. Additionally, this cone subtype is the only one for which a transgenic line, expressing mKate-tagged opsin and thus marking the OS, is available [TgBac(opn1lw1:opn1lw1-mNeonGreen/opn1lw2:opn1lw2-mKate, referred to as Tg(LWS)] (Crespo et al., 2018). This transgenic line, in combination with phalloidin staining, allowed for clear visualisation and quantification of IS and OS size of LWS cones. The IS was defined as the region between the junction, marked by phalloidin, and the base of the OS, marked by mKate2. The clear signal of mKate-opsin allowed for reliable segmentation and volume quantification of OS. At 72 hpf, no significant differences were observed in OS volume and IS length at the different light conditions (Fig. 6A). By 120 hpf, ISs of LWS cones showed a significant increase in length under all light conditions. However, the degree of increase was dependent on light. While in constant darkness IS length increased by an average of 81%, IS length of PRCs exposed to light/dark cycles or constant light only increased in length by an average of 77% and 60%, respectively (Fig. 6A). A similar influence of light was observed with respect to OS volume. For embryos exposed to constant light, no obvious OS increase was observed (Fig. 6A). In contrast, for embryos raised either in constant darkness or in light/dark cycles, an increase in OS volume was observed. While OSs of embryos raised in constant darkness showed an average increase in volume of 296%, those exposed to light/dark cycles only increased by 104%. This suggests that light exposure could significantly delay apical membrane growth during PRC maturation. Alternatively, this could be also due to changes in OS discs shedding.

Interestingly, we observed a striking change in transgene-encoded opsin distribution under constant light exposure. While opsin is restricted to the OS in the dark and under dark/light conditions, strong accumulation of fluorescent clusters of tagged red opsin were observed in the cell body upon exposure of embryos to constant light (Fig. 6B). Further analyses are required to determine whether the delay in apical membrane growth is causally related to opsin misdistribution.

## DISCUSSION

### Criteria to define maturation stages and PRC subtypes

The goal of this work was an in-depth characterisation of subtype-specific maturation of zebrafish PRCs. To this end, we quantified the size of individual cellular compartments, the localisation of polarity proteins and the positioning of organelles during early stages of PRC maturation, i.e. from about 51 hpf to 120 hpf. One of the observations of this study was the anisotropic and subtype-specific growth of PRC compartments (Table S1). After the emergence of the OS, the ISs show an overall growth over-time in all PRC subtypes, with the exception of the UVS cones, in which the IS length decreases. This is in contrast to the basal sides, which shows an obvious decrease in all PRCs by 96 hpf. One may speculate that this reduction could be the result of the folding of the membrane on the basal side during synapse development (Schmitt and Dowling, 1999). The OS of all PRC subtypes is the compartment with the highest growth rate over time. Interestingly, the smallest ISs were found in those cones that had the largest OSs. This inverse correlation could be important for the OS to reach the retinal pigment epithelium, which is essential for PRC function and maintenance.

**Fig. 7.**
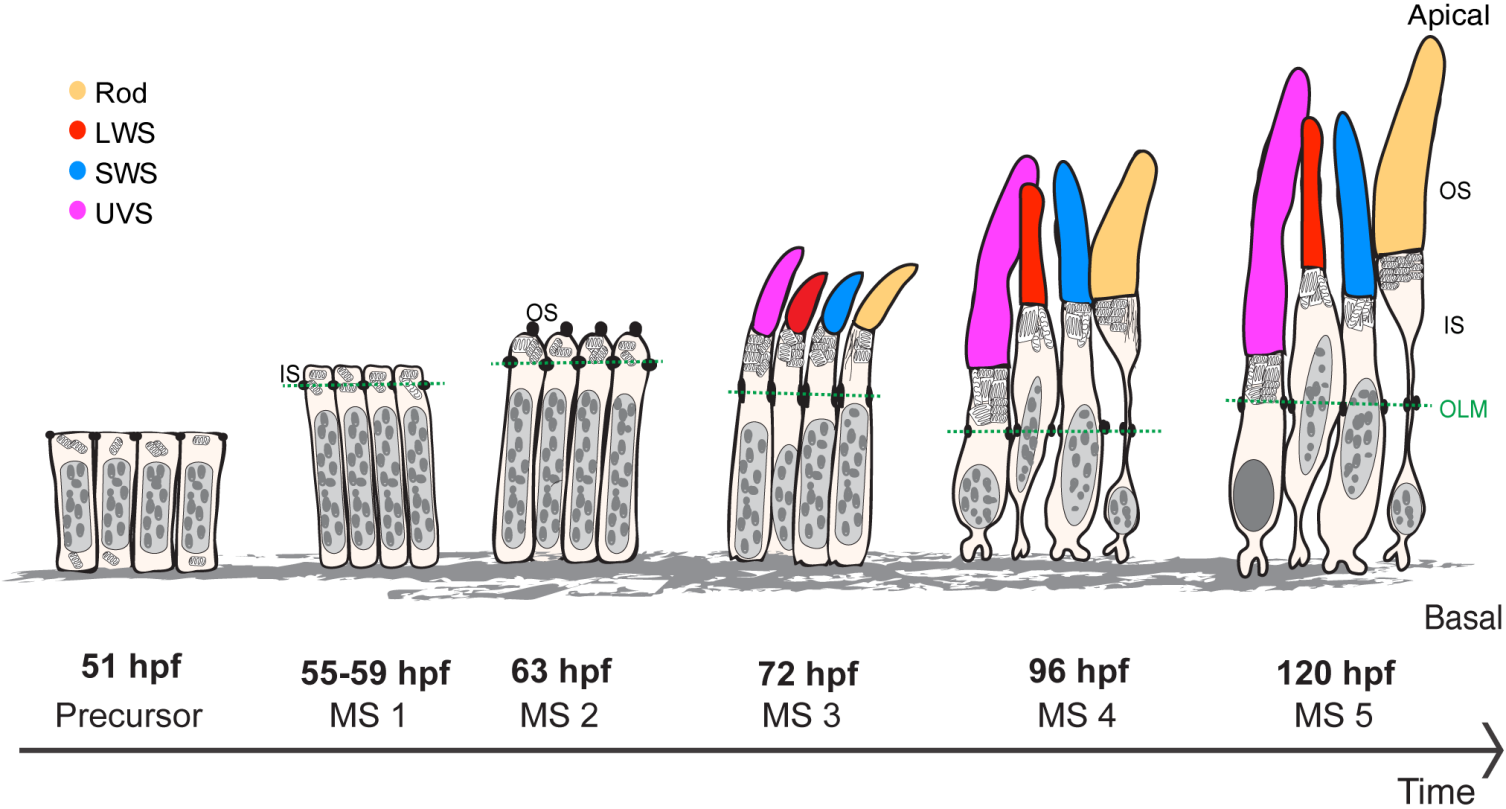
**Maturation of PRCs, from precursor to 120 hpf.** Different stages of PRC maturation during zebrafish retinal development. OS of the different PRC subtypes are highlighted in magenta (UVS cones), yellow (rods), blue (SWS cones) and red (LWS cones). This schematic depicts the size of the cell body, IS and OS height and OS width (the same arbitrary scale is used for all stages, with the exception of the synapses). Nuclei are represented in grey (eu- and heterochromatin represented in light and dark grey, respectively). Mitochondria and ER (in the IS of rods at stage 3 and 4) are shown in the IS of PRCs. OLM is marked by a green dash line.

The other important finding of this work was the identification of cellular features, which can be used to distinguish PRC subtypes from early stages on. In addition, the data led us to define phenotypic criteria allowing a more precise retinal staging, which is necessary when the developmental age of zebrafish embryos cannot be used, e.g. in certain mutants. In the following paragraphs, we will elaborate on these two features during maturation (Fig. 7; Table S1).

From the precursor stage (51 hpf) to the first detection of the OS by immunohistochemistry (63 hpf), no obvious differences were observed among PRC subtypes in terms of morphology, growth, organelle positioning and chromatin organisation. No subdivision of the apical membrane is obvious. Maturation during this period is characterised by an expansion of the apical side only, while no significant changes are observed in the basal side of the cells. Interestingly, the initially rapid growth of the IS slows down at the time when OS are first detected by immunohistochemistry. The discrepancy in the timepoint of OS appearance described here (63 hpf) and in an earlier study (55 hpf) (Schmitt and Dowling, 1999) could be explained by differences in the sensitivity of both techniques and minor differences in positioning the region of interest, which thus would contain PRCs at slightly different maturation stages due to the wave-like differentiation of the retina.

As early as 72 hpf, rods are the first PRCs that can be distinguished from the other PRCs by the transient accumulation of rough ER in the ellipsoid, a region that becomes distinct from the myoid area around the same time when the OSs appear (Kim et al., 2005). The transient accumulation of ER in rods is not only a suitable tool to identify this particular PRC subtype at this stage, but its transient nature can also be used for determining the maturation stage. The temporal coincidence of the onset of OS formation and ER accumulation in the ellipsoid of rods let us to speculate that this may be due to meet the increased demand of protein and lipid biosynthesis required for OS membrane formation of rods, one of the PRC subtypes with the largest growth.

Nuclei and mitochondria positioning provides useful criteria for defining maturation stages after 72 hpf. Up to 72 hpf, all nuclei occupy a central position in each cell, thus forming a single row of nuclei in the retina. From 96 hpf onwards, two separate nuclear rows are visible, which is more pronounced at 120 hpf. Similarly, two independent rows of mitochondria are observed by 96 hpf, which resolve into three rows at 120 hpf. This is in accordance with previous studies, which described mitochondria in different positions from 96 hpf onwards (Tarboush et al., 2014). Moreover, Tarboush’s study reported differences in mitochondria size between rods (small mitochondria) and cone PRCs (megamitchondria) from 96 hpf, which can also be used as a marker for rod PRCs (Tarboush et al., 2014).

At 120 hpf, all four PRC subtypes can be distinguished. UVS cones can be defined at this stage by having more homogenously dispersed heterochromatin. This is comparable to the inverted chromatin organisation observed in mouse rods (Solovei et al., 2009). In biological tissues, the degree of light scattering depends on the wavelength of the light; the lower the wavelength, the more light scattering occurs (Jacques, 2013). Therefore, we hypothesise that changes in chromatin organisation of UVS cone nuclei could help to reduce light scattering during development by acting as a lens that channels light in the UVS OS, a function similar to that suggested for the inverted chromatin shown in mouse rods (Solovei et al., 2009). At 120 hpf the different PRC subtypes can additionally be distinguished by differences in IS and OS size. UVS cones show the shortest IS length, PRCs showing the largest width of the OS could be defined as rods, and UVS cones have the longest OS, followed by rods, SWS and LWS cones.

Based on our data, we would suggest to define six stages of PRC maturation, from precursors to 120 hpf, namely PRC precursor stage and maturation stage (MS) 1–5 (Fig. 7; Table S1). The first three stages of PRC maturation (PRC precursor, MS1 and MS2) were categorised based on cell morphology. The PRC precursor stage is characterised by cells with a columnar morphology and the absence of an obvious subdivision of the apical side. MS1 begins with the growth of the apical side and the appearance of the sub apical region (SAR) defined by the presence of Crb2a. This stage is mainly characterised by the growth of the presumptive inner segment (IS). MS2 is characterised by the onset of outer segment (OS) growth and expression of Crb2b. In these initial three stages (PRC precursor, MS1 and MS2) no morphological differences are observed among different subtypes of PRCs. MS3, MS4 and MS5 were defined as independent maturation stages based on a combination of morphological and cellular traits (e.g. growth rates of different cell compartments, morphological differences among different subtypes of PRCs, and distribution of cell organelles). These stages are of particular interest because they have been widely investigated in previous studies of zebrafish retinal development (Krock and Perkins, 2014; Raghupathy et al., 2016; Tarboush et al., 2014). At MS3, nuclei show small changes in their shape and positioning; mitochondria of all PRC subtypes are perfectly aligned in the PRC layer, forming a single row, and rods show an accumulation of ER. At MS4, two rows of mitochondria and nuclei become visible in the PRC layer. Moreover, changes in OS length, a decrease in the IS of UVS cones, and a striking decrease of the basal side of PRCs occur. By MS5, mitochondria localised in the ellipsoid of all PRCs appear in three rows. Taking together the average of IS length of the different PRCs and previous reports on the zebrafish retina (Branchek and Bremiller, 1984; Fang et al., 2017; Schmitt and Dowling, 1999), we hypothesise that the upper mitochondrial row corresponds to LWS cones together with rods, the second to SWS cones and the third to UVS cones. Additionally, UVS cones show alterations in chromatin organisation, and striking differences in OS and IS length are observed in PRC subtypes.

### Effect of prolonged exposure to light on PRC maturation

The zebrafish is ideally suited to study retinal development and has turned out as a useful model to unravel the cellular and molecular basis of human retinal diseases (Gestri et al., 2012; Link and Collery, 2015; Raghupathy et al., 2013). However, genetic and environmental perturbations often also affect the developmental rate. For example, retinoic acid results in a delay of cone maturation (Hyatt et al., 1996). Therefore, a staging scheme is required, which allows embryos to be staged according to their maturation age, independent of the time elapsed since fertilisation. Having established a detailed timeline for PRC maturation in wild-type zebrafish embryos, we applied this knowledge to analyse the influence of light on the maturation of LWS cones. Previous reports already demonstrated light-dependent effects on IS and OS size in different vertebrates and even flies (Abràmoff et al., 2013; Hodel et al., 2006; Pocha et al., 2011). At about 72 hpf, no significant changes were observed in IS and OS length of embryos exposed to different light conditions (constant light, light/dark cycle and constant dark). However, when embryos were grown under light/dark cycles for 120 hpf, IS and OS length had a lower increase compared to their siblings raised in constant darkness. This phenotype was even more pronounced when embryos were raised in constant light. The changes in IS length could be the result of changes in the myoid elongation. On the other hand, changes in OS growth of embryos exposed to constant light could be explained by the mislocalisation of a substantial portion of opsin in cell bodies of LWS cones, which was very rarely detected under the other two light conditions. Previous findings demonstrated that zebrafish embryos exposed to constant light show a decrease in red opsin mRNA expression (Li, 2005). So we cannot exclude the possibility that a decrease in red opsin protein due to decreased red opsin mRNA may contribute to decreased OS growth under constant light. Alternatively, the changes in OS volume could be due to changes in discs shedding, which is influenced by light/dark cycles (Basinger and Hollyfield, 1980; Besharse and Hollyfield, 1979; Kocaoglu et al., 2016; Young, 1967).

Mislocalisation of opsin has been linked to retinal degeneration, both in vertebrates and flies (Hollingsworth and Gross, 2012; Lu et al., 2017; Pocha et al., 2011; Raghupathy et al., 2017; Xu and Wang, 2016). However, the fact that the IS still grows under constant light conditions suggests that PRCs are still maturing.

To conclude, the extensive characterisation of PRC subtype maturation in the zebrafish retina provides a platform for further studies of retinal development and disease. In addition, our work highlights the importance of carefully controlling light conditions under which the fish are raised, and thoroughly paying attention to identify PRC subtypes when performing quantitative analyses of PRC maturation.

## MATERIALS AND METHODS

### Animal husbandry

Adult zebrafish were maintained at 28°C under standard conditions in a 14 h on/10 h off, light/dark cycle. All embryos were born and collected between 10 am and 11 am. Up to 120 hpf, embryos were raised in E3 medium in a dark incubator at 28.5°C, with exception of those used for the studies of the influence of light on PRC maturation, which were raised either in constant light (∼500 lux) and in light/dark cycles (14 h light, 500 lux, and 10 h dark). In light/dark cycle, the light was on from 7 pm to 9 am. Zebrafish lines used in this study were: wild-type (WT) AB, Tg(Ola.Actb:Hsa.HRAS-EGFP)^vu119^ (Cooper et al., 2005), Tg(EF-1α:MLS-EGFP) (Kim et al., 2008), (Tg(-5.5opn1sw1:EGFP) (Takechi et al., 2003), Tg(opn1lw1:opn1lw1-NeonGreen/opn1lw2:opn1lw2-mKate) (Crespo et al., 2018). All animal studies were performed in accordance with European and German animal welfare legislation. Protocols were approved by the Institutional Animal Welfare Officer (Tierschutzbeauftragter), and necessary licenses were obtained from the regional Ethical Commission for Animal Experimentation of Dresden, Germany (Tierversuchskommission, Landesdirektion Sachsen).

### Microinjections of plasmid

30 pg of plasmid rasmKate (Weber et al., 2014) was injected into 1-cell-stage WT embryos using a PV820 Pneumatic PicoPump Injector (World Precision Instruments, USA). For the injections, glass 1.0 mm capillaries (World Precision Instruments, USA) were pulled to create injection needles.

### Electron microscopy – sample preparation

Heads of zebrafish embryos at defined stages were dissected and fixed with 2% PFA (paraformaldehyde), 2% glutaraldehyde in 0.1 M Hepes buffer for 1 h at room temperature (RT) and then overnight at 4°C. Samples were washed in 0.1 M Hepes buffer (5×3 min) and then in 1xPBS. 1% OSO_4_, 1.5% potassium ferrocyanide in PBS was added to the samples and incubated at RT for 90 min. Samples were washed in water and dehydrated in a series of EtOH (30%, 50%, 70%, 90% and 100%, for 20 min in each solution). Samples were incubated at 60°C in 100% propoxide (prop.Oxide) (Sigma-Aldrich) for 20 min, 2:1 prop.Oxide:Durcupan (Sigma-Aldrich) for 1 h, 1:1 prop.Oxide:Durcupan for 1.5 h and finally 1:2 prop.Oxide:Durcupan overnight. Samples were then incubated in 100% Durcupan (Sigma-Aldrich) overnight. Fresh Durcupan was added after the first 5–6 h of incubation. Samples were mounted in 100% Durcupan and sectioned for electron microscopy (0.1 mm thickness). Before imaging, all TEM samples were stained with 2% uranyl acetate (Polysciences, Inc.) in water for 10 min, followed by 5 min in lead(II) citrate tribasic trihydrate (Sigma-Aldrich).

### Crysectioning ­– sample preparation

Whole embryos up to 120 hpf were fixed using 4% PFA in PBS for 1 h at RT, with the exception of embryos used for opsin stainings, which were fixed in 37% PFA:100% ethanol (EtOH)(1:9). Samples fixed in EtOH were rehydrated after fixation, 10 min 90% EtOH, 10 min 80% EtOH, 10 min 70% EtOH, 10 min 50% EtOH, 10 min PBS. After fixation, all samples were washed for 2×5 min in PBS and kept for 1 h in 5% sucrose in 1xPBS, and then incubated overnight at 4°C in 30% sucrose in 1xPBS, followed by a second overnight incubation at 4°C in 1:1 solution of 30% sucrose in 1xPBS:NEG-50™ (Thermo Fisher Scientific). Finally, the samples were incubated for 1 h at RT in NEG-50™, mounted and frozen in dry ice. All samples were kept at −80°C until sectioning. 16 µm sections of zebrafish eyes were left to dry for at least 1 h at room temperature. After sectioning, all samples were kept at −20°C until use.

### Visualization of endogenous fluorescence from transgenic animals

Before imaging, retinal sections were dried for at least 1 h at room temperature followed by rehydration in 1xPBS for 30 min. For nuclear staining, sections were incubated for 10 min in DAPI diluted 1:10,000 (Thermo Fischer Scientific) and Phalloidin 660, diluted 1:20 (Thermo Fisher Scientific A22285) in PBS at RT. Ultimately, sections were washed 3 times for 5 min at RT and mounted in Vectashield antifade mounting medium (Vectorlabs).

### Generation of Crb2b antibody

Rat polyclonal antibodies against the long isoform of Crb2b (Crb2b-lf) (Zou et al., 2012) were generated by the injection of Crb2b-lf peptide into two rats, rat 1 and rat 2. The used peptide sequence of Crb2b was MRGLIVKVICCGLLLLTGAVCETELDECESDPCQNRGRCEDSINAYICHCPPAEPGHLPWGGPDCSVQLTG. The peptide was generated by Peptide Specialty Laboratories GmbH, Heidelberg. Peptides were sent to Charles Rivers, Germany, for injection of rats. Antibody specificity was investigated by detection of Crb2b-lf by western blot (data not shown) and immunohistochemistry in retinal sections.

### Immunohistochemistry

Cryosections were left to dry at RT for 1 h prior to staining. Slides were washed in PBS for 20 min at RT. For Crb2a and Crb2b antibodies, an extra step of permeabilization was done (0.1% SDS for 15 min+3×5 min in PBS). Blocking of samples was done using 10% normal horse serum (NHS) in 0.3% Triton X-100 in PBS. Primary antibodies were diluted in blocking solution and incubated overnight at 4°C. Opsin antibodies were incubated twice overnight at RT. The following primary antibodies were used: rabbit polyclonal anti-PrkC (Abcam ab19031; 1:50), mouse monoclonal anti-Crb2a (ZIRC; 1:50), rabbit polyclonal anti-opsin (1:50) (Vihtelic et al., 1999), rat anti-Crb2b (this study, rat 2, 1:200), mouse monoclonal anti-ZO-1 (Sigma AB2271; 1:200) and rabbit polyclonalanti-H3K4me3 (Diagenome, C15410003-50; 1: 200). Slides were washed 3× for 20 min in PBST (0.1% Triton X-100 in PBS) and incubated with secondary antibody in combination with DAPI, diluted 1:10,000 in blocking solution overnight at 4°C. Secondary antibodies were Alexa Fluor anti-mouse 568, anti-rat 568 and anti-rabbit 568 from Thermo Fisher Scientific (1:500). Phalloidin 660 (Thermo Fisher Scientific A22285; 1:20) was incubated together with secondary antibodies. Slides were washed 2×20 min in PBST, 2×20 min in PBS and finally mounted in Vectashield antifade mounting medium (Vectorlabs). Slides were kept at 4°C until imaging.

### Imaging and image analysis

All samples immunoassayed were imaged using a ZEISS multiphoton laser scanning upright microscope (model Axio Examiner.Z1), with a Zeiss Plan-Neofluar 63× NA 0.8 objective. All images were acquired using ZEN 2011 software (black edition) from ZEISS. Electron microscopic images were acquired using Morgani 268 microscope (Phillips). iTEM software (ResAlta Research Technologies) was used for capturing the images. All image analyses and length quantifications were done using Fiji (Schindelin et al., 2012). For volume measurements, OS were segmented, selected and quantified using Imaris 7.7 software (Bitplane, Oxford Instruments). Growth percentages were calculated with the following formula: growth %=(average length at 120 hpf- average length at 72 hpf)/ average length at 72 hpf)*100. The graphs were plotted and statistical analyses were done with GraphPad Prism6. Statistical significance was calculated by a one-way or two-way ANOVA followed by a Tukey's multiple comparison test. Individual details are given in the figure legends of the respective graphs.

## Supplementary Material

Supplementary information
