# Supplementary material for: Characterisation of maturation of photoreceptor cell subtypes during zebrafish retinal development
Source: Biol Open. 2018 Sep 20;7(11):bio036632. doi: 10.1242/bio.036632 (PMC6262866; doi:10.1242/bio.036632)
Supplement: Supplementary information [file biolopen-7-036632-s1.pdf]

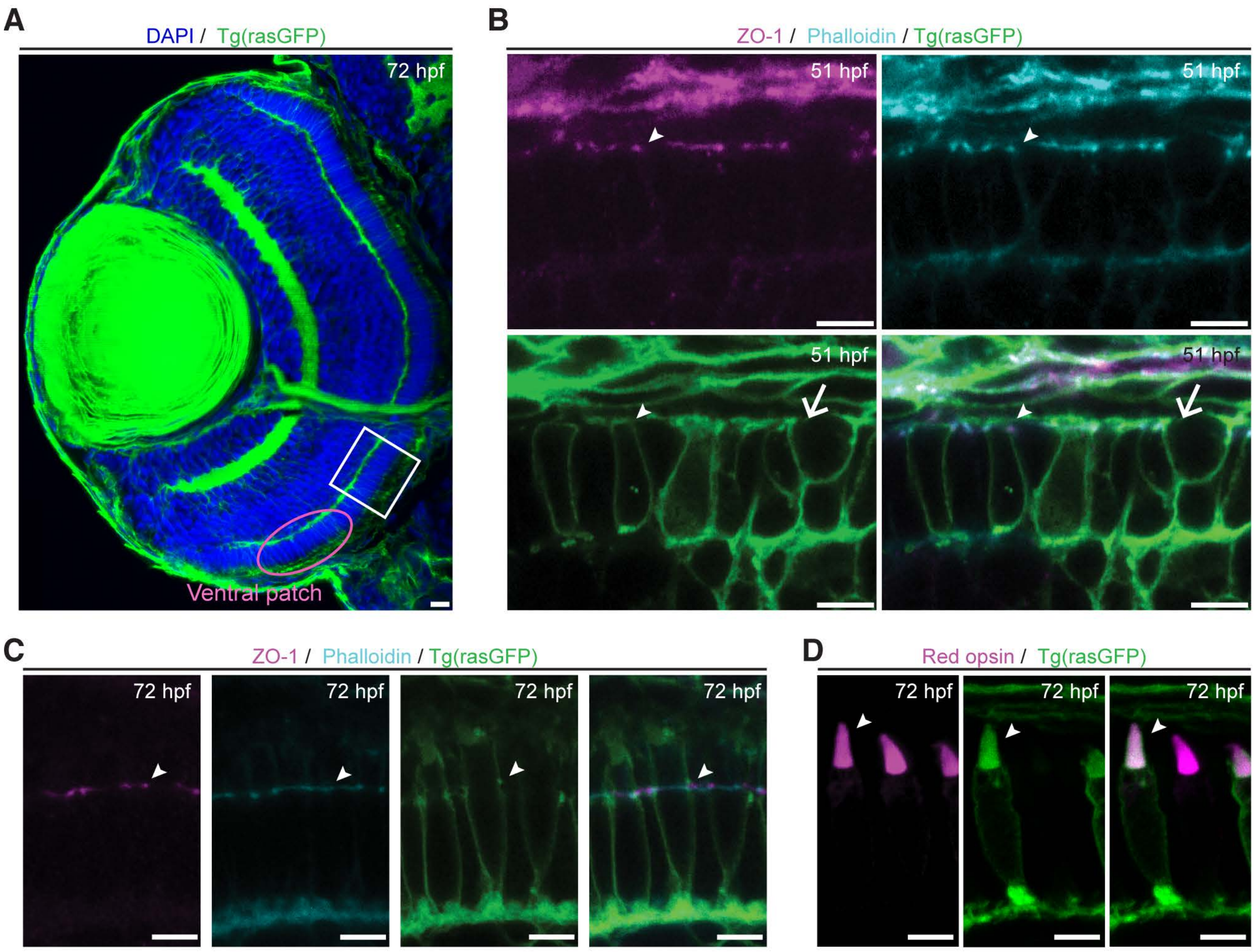

Figure S1. - Tg(rasGFP) line as a tool to identify PRC compartments.

A-D: Retinal section of zebrafish Tg(rasGFP) embryos at 72 hpf, with endogenous fluorescence of membranes marked in green. **A:** Confocal image of the full zebrafish eye section with DAPI in blue. The white box ventral to the optic nerve demarcates the region from which the images shown in Fig. 1 were taken, and the magenta circle demarcates the ventral patch. **B:** Confocal image of PRCs stained with ZO-1 (magenta) and phalloidin (cyan). Arrowheads highlight junctions and arrows point to dividing cell. **C:** Fluorescent structures marked by Tg(rasGFP) (green) co-localise with ZO-1 (magenta) and phalloidin (cyan) (arrowheads), thus highlighting the junction. **D:** Endogenous fluorescence of TgBac(opn1lw1:opn1lw1-mNeonGreen/opn1lw2:opn1lw2-mKate) (magenta) highlights the OS, which overlaps with the cone-shaped structure marked by Tg(rasGFP). Arrowhead highlights OSs. Scale bars: A: 10 μm, B-D: 5 μm.

Biology Open • Supplementary information

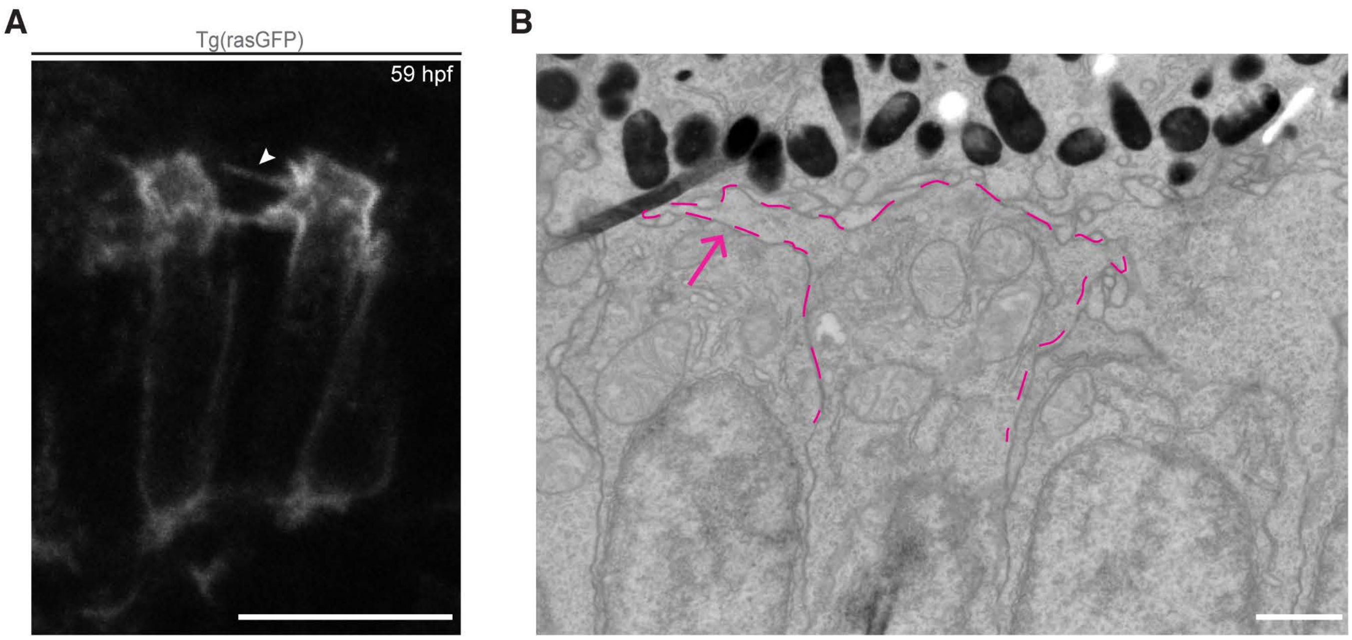

Figure S2. - Formation of processes in the growing IS of maturing PRCs.

A: Visualisation, by confocal imaging, of retinal sections of embryos at 59 hpf expressing the Tg(rasGFP) construct (plasma membrane marker). Processes (arrowhead) appear at the same time as the IS becomes visible and are still visible when OSs form. **B:** Electron micrograph of a section through the PRC layer in zebrafish embryo aged 55 hpf. The magenta dashed line highlights a process extending from the growing IS (magenta arrow). Scale bars: A: 10 μm, B:1 μm.

Biology Open • Supplementary information

Table S1 – Summary of the different features associated with the different PRC maturation stages.

Feature	Stage of development					
	PRC Precursors ~ 51 hpf	Stage 1 ~55-59 hpf	Stage 2 ~63 hpf	Stage 3 ~72 hpf	Stage 4 ~96 hpf	Stage 5 ~120 hpf
**Growth of PRC compartments**						
Cell body (^{min} average length ^{max} [µm])						
Tg(rasGFP)	$^{8.1}$ 9.7 $^{11.1}$	$^{10.3}$ 12.7 $^{15.0}$	$^{11.6}$ 13.8 $^{15.3}$	$^{10.8}$ 14.5 $^{17.7}$	$^{9.7}$ 13.6 $^{18.0}$	$^{8.5}$ 13.1 $^{19.8}$
UVS	-	-	-	$^{12.2}$ 14.4 $^{16.3}$	$^{9.7}$ 12.5 $^{15.7}$	$^{8.5}$ 10.8 $^{13.7}$
SWS	-	-	-	$^{12.1}$ 15.3 $^{18.1}$	$^{14.9}$ 16.6 $^{17.6}$	$^{15.3}$ 17.2 $^{19.2}$
LWS	-	-	-	$^{12.3}$ 15.0 $^{20.0}$	$^{12.5}$ 16.4 $^{20.3}$	$^{13.1}$ 18.2 $^{22.6}$
Rod	-	-	-	$^{13.5}$ 15.1 $^{17.2}$	$^{14.7}$ 17.2 $^{21.5}$	$^{15.7}$ 18.8 $^{23.7}$
Basal side (^{min} average length ^{max} [µm])						
Tg(rasGFP)	-	$^{9.2}$ 10.7 $^{12.2}$	$^{11.5}$ 12.1 $^{12.7}$	$^{7.5}$ 10.3 $^{12.4}$	$^{6.5}$ 8.2 $^{9.8}$	$^{7.5}$ 9.1 $^{11.9}$
Inner segment (^{min} average length ^{max} [µm])						
Tg(rasGFP)	-	$^{0.4}$ 1.7 $^{2.7}$	$^{1.2}$ 1.9 $^{3.2}$	$^{1.2}$ 3.3 $^{5.7}$	$^{1.9}$ 4.4 $^{8.5}$	$^{1.0}$ 4.8 $^{11.4}$
UVS	-	-	-	$^{2.0}$ 3.1 $^{4.4}$	$^{2.5}$ 3.8 $^{6.6}$	$^{1.1}$ 2.7 $^{5.1}$
SWS	-	-	-	$^{1.9}$ 2.9 $^{4.3}$	$^{4.8}$ 5.7 $^{6.7}$	$^{6.5}$ 8.1 $^{9.0}$
LWS	-	-	-	$^{1.0}$ 3.5 $^{8.1}$	$^{3.1}$ 5.6 $^{8.1}$	$^{6.6}$ 8.2 $^{10.3}$
Rod	-	-	-	$^{2.0}$ 3.1 $^{4.6}$	$^{4.3}$ 6.3 $^{8.9}$	$^{5.6}$ 8.9 $^{12.6}$
Outer segment (^{min} average length ^{max} [µm])						
Tg(rasGFP)	-	-	$^{0.2}$ 0.6 $^{0.8}$	$^{0.6}$ 3.8 $^{8.2}$	$^{3.7}$ 7.9 $^{13.1}$	$^{2.9}$ 10.2 $^{16.5}$
LWS	-	-	-	$^{0.2}$ 1.2 $^{3.3}$	$^{2.5}$ 4.4 $^{6.1}$	$^{4.7}$ 5.9 $^{8.1}$
SWS	-	-	-	$^{0.7}$ 1.7 $^{2.9}$	$^{3.2}$ 5.2 $^{9.4}$	$^{5.3}$ 7.7 $^{10.4}$
Rod	-	-	-	$^{0.9}$ 2.6 $^{5.6}$	$^{3.8}$ 5.3 $^{8.1}$	$^{6.2}$ 8.3 $^{10.2}$
UVS	-	-	-	$^{2.3}$ 5.3 $^{8.2}$	$^{6.4}$ 8.8 $^{13.1}$	$^{10.3}$ 12.7 $^{16.5}$
Outer segment (^{min} average length ^{max} [µm])						
LWS	-	-	-	$^{0.8}$ 1.2 $^{1.8}$	$^{1.0}$ 1.5 $^{1.9}$	$^{1.2}$ 1.6 $^{2.3}$
SWS	-	-	-	$^{1.0}$ 1.3 $^{2.0}$	$^{1.1}$ 1.5 $^{2.0}$	$^{1.6}$ 2.1 $^{3.2}$
UVS	-	-	-	$^{1.0}$ 1.3 $^{1.7}$	$^{1.0}$ 1.8 $^{2.7}$	$^{1.6}$ 2.3 $^{3.1}$
Rod	-	-	-	$^{0.9}$ 1.6 $^{2.8}$	$^{1.0}$ 2.1 $^{3.0}$	$^{2.0}$ 2.7 $^{3.4}$
**Cell organelles**						
Mitochondria localisation	-	Dispersed in the apical domain	In ellipsoid region in some PRCs	Aligned in 1 row (ellipsoid)	Aligned in 2 adjacent rows (ellipsoid)	Aligned in 3 rows (ellipsoid)
Rough ER clusters	-	-	-	Only in Rod PRCs		-
Nuclei position in ONL	Aligned in one row (rectangular)			Initial changes in positioning	Obvious separation into 2 rows	
Chromatin organisation	Conventional Chromatin in all PRC subtypes					Changes in UVS cones
**Polarity Proteins**						
PrkC	Apical membrane			Sub apical region (SAR)		
Crb2a	Apical membrane	Sub apical region (SAR)				
Crb2b	-	-	Sub apical region (SAR)			

Biology Open • Supplementary information
